# Delayed Feeding Alters Transcriptional and Post-Transcriptional Regulation of Hepatic Metabolic Pathways in Peri-Hatch Broiler Chicks

**DOI:** 10.3390/genes10040272

**Published:** 2019-04-03

**Authors:** Julie A. Hicks, Tom E. Porter, Nishanth E. Sunny, Hsiao-Ching Liu

**Affiliations:** 1 Department of Animal Science, North Carolina State University, Raleigh, NC 27607, USA; jahicks3@ncsu.edu; 2 Department of Animal and Avian Sciences, University of Maryland, College Park, MD 20742, USA; teporter@umd.edu (T.E.P.); nsunny@umd.edu (N.E.S.)

**Keywords:** chicken, metabolic switch, microRNA

## Abstract

Hepatic fatty acid oxidation of yolk lipoproteins provides the main energy source for chick embryos. Post-hatching these yolk lipids are rapidly exhausted and metabolism switches to a carbohydrate-based energy source. We recently demonstrated that many microRNAs (miRNAs) are key regulators of hepatic metabolic pathways during this metabolic switching. MiRNAs are small non-coding RNAs that post-transcriptionally regulate gene expression in most eukaryotes. To further elucidate the roles of miRNAs in the metabolic switch, we used delayed feeding for 48 h to impede the hepatic metabolic switch. We found that hepatic expression of several miRNAs including *miR-33*, *miR-20b*, *miR-34a*, and *miR-454* was affected by delaying feed consumption for 48 h. For example, we found that delayed feeding resulted in increased *miR-20b* expression and conversely reduced expression of its target *FADS1*, an enzyme involved in fatty acid synthesis. Interestingly, the expression of a previously identified *miR-20b* regulator *FOXO3* was also higher in delayed fed chicks. FOXO3 also functions in protection of cells from oxidative stress. Delayed fed chicks also had much higher levels of plasma ketone bodies than their normal fed counterparts. This suggests that delayed fed chicks rely almost exclusively on lipid oxidation for energy production and are likely under higher oxidative stress. Thus, it is possible that FOXO3 may function to both limit lipogenesis as well as to help protect against oxidative stress in peri-hatch chicks until the initiation of feed consumption. This is further supported by evidence that the FOXO3-regulated histone deacetylase (*HDAC2*) was found to recognize the *FASN* (involved in fatty acid synthesis) chicken promoter in a yeast one-hybrid assay. Expression of *FASN* mRNA was lower in delayed fed chicks until feed consumption. The present study demonstrated that many transcriptional and post-transcriptional mechanisms, including miRNA, form a complex interconnected regulatory network that is involved in controlling lipid and glucose molecular pathways during the metabolic transition in peri-hatch chicks.

## 1. Introduction

During embryonic development, chicks utilize yolk lipids for energy. During the last stages of development, the embryo absorbs the yolk sac, which continues to provide a residual lipid source during the first few days post-hatch [1]. Following feed consumption after hatching, the chick’s metabolism then must switch to a mainly carbohydrate-based energy source. Therefore, during embryogenesis, hepatic expression of genes associated with lipid oxidation is high, and then post-hatching hepatic lipogenic gene expression increases as feed consumption begins. Glucose levels increase after hatch, resulting from both increased breakdown of hepatic glycogen stores as well as increased glucose production from a carbohydrate rich diet [2]. Delayed feeding of chicks for 48 h also delays the induction of hepatic lipogeneic gene expression [3].

Ketone bodies have been shown to be highly produced during the latter half of embryonic life and in the newly hatched chick (reviewed by [4]). These ketone bodies are generated in the liver from the oxidative break down of yolk triglyceride-derived fatty acids. This high production of ketone bodies is thought to provide fuel for other tissues, particularly muscle, to reserve the limited in ovo carbohydrates for biosynthetic processes. Upon feed consumption, ketone bodies are still generated from hepatic triglycerides, but their levels quickly drop. The increased utilization of ketone bodies by late term/early hatch chicks is mainly due to the high levels of ketone bodies rather than major differences in enzymatic activity [5].

During the last stages of embryonic brain development there is a significant accumulation of cholesterol, which is associated with ketone body-associated lipid synthesis [5]. This high cholesterol production during late embryogenesis is mainly due to LPL-mediated lipolysis of yolk VLDL triglycerides [2]. Embryonic chick hepatocytes, particularly from birds near hatching, have large lipolysomes mainly (76%) consisting of esterified cholesterol [6]. It was found that these lipolysome-associated lipid stores are quickly depleted upon feed intake, and fasting retards lipolysis of these stores as well as lipoprotein transport [6]. Cholesterol levels drop substantially upon feeding but remain at higher levels in delayed fed chicks [6].

In vertebrates, small regulatory non-coding RNAs termed microRNAs (miRNAs) regulate many diverse metabolic processes. In humans and rodents, *miR-27b* was shown to regulate a number of lipid metabolism-associated genes, including *GPAM* and *ANGPLT3* [7]. Hepatic expression of *miR-27b* was higher in mice receiving a high fat diet. It was suggested that *miR-27b* may serve to reduce lipid accumulation [7]. One well-established metabolic miRNA is *miR-33*. Several studies have demonstrated *miR-33* regulation of cholesterol synthesis and transport [8,9]. Of note, *miR-33* is located in an intron of *SREBF2*, which encodes for a transcriptional regulator of cholesterol synthesis [8]. It was also shown that *miR-33* levels in the liver are negatively correlated with cholesterol levels. Inhibition of *miR-33* function in African green monkeys led to increased fatty acid oxidation and a decrease in fatty acid synthesis [10].

In addition to regulating lipid metabolism, miRNAs also regulate a number of glucose metabolic pathways. Interestingly, in a diabetic mouse model, a number of lipid metabolic genes including, *PPARGC1A*, *HMGCS2*, and *ADDHD5*, had altered expression, which was attributed to *miR-29a* regulation [11]. In mammals, it has been well established that miRNAs are involved in most cholesterol metabolic processes. The miRNA *miR-30c* regulates microsomal triglyceride transfer protein (MTP) which reduces plasma cholesterol levels. The miRNAs *miR-130b* and *miR-301b* can both target the hepatic glucogenesis regulator SIK1 (reviewed by [12]). *MiR-130b* can also regulate PPARGC1A and INSIG1 levels, altering cholesterol synthesis and transport. A large GWAS analysis identified SNPs for 69 miRNAs associated with metabolic disorders [13]. Among these is *miR-148a*, which regulates several lipid and cholesterol homeostatic genes including *LDLR*, *ABCA1*, and *SIK*.

We recently discovered that a complex miRNA regulatory network likely helps mediate the metabolic transition from embryonic to post-hatch life in chickens [14]. We found that miRNAs likely regulate a number of major metabolic genes, including *INSIG1* and *SREBF1*, among others. Here, we further characterized these metabolic miRNAs in delayed fed broiler chicks. We found that several metabolic pathways involved in lipogenesis, lipolysis and carbohydrate metabolism were affected by delaying feeding of male broilers for 48 h. We also found that miRNA mediators of these pathways were also affected. This suggests that miRNAs are indeed important regulatory factors of the hepatic metabolic switch in peri-hatch chicks.

## 2. Materials and Methods

### 2.1. Birds

All animal procedures were approved by North Carolina State University’s IACUC (protocol #17-179-A). Fertilized Ross 708 eggs were obtained from Perdue Farms Hatchery (Hurlock, MD, USA) and incubated under standard conditions. On embryonic day (E)18, eggs were weighed and eggs near the mean (57 g ± 2) were transferred to a hatchery. Birds hatching between 6:00 and 12:00 on E21/day of hatch (D)0 were feather sexed and weighed. Males weighing between 46 g and 48 g were randomly divided into two groups. Birds in each group were then randomly divided among 6 cages (15 birds per cage). The first group (control) received chick starter feed (Southern States, Richmond, VA, USA; Table S1) *ad libitum* on D0. Feeding of the second group (delayed) was delayed for 48 h, and on D2 the second group received chick starter feed (Southern States) *ad libitum*. All birds were allowed *ad libitum* access to water from D0.

### 2.2. Sample Collection

On E18, E20, D0, D1 (control and delayed), D2 (control and delayed), D3 (control and delayed), D4 (control and delayed), and D7 (control and delayed), birds were weighed, and plasma and liver samples were collected from six males in each group (one male per cage). Sex was confirmed by gonad identification. For embryonic birds the entire left lobe of the liver was snap frozen, for post-hatch birds approximately one gram of the left lobe was snap frozen. All liver samples were stored at −80 °C until analyzed.

### 2.3. Free Fatty Acids Assay

Plasma free fatty acids were measured using a Free Fatty Acid Fluorometric assay kit (Cayman Chemical, Ann Arbor, MI, USA), following the manufacturer’s instructions. Briefly, plasma from six males in each group was assayed in triplicate. To 10 µL of plasma, 200 µL of FFA cofactor mixture was added and incubated for 30 min at 37 °C. Then, 100 µL of developer was added and incubated at 37 °C for 15 min. Fluorescence was measured on a BioTek Synergy HT plate reader (BioTek, Winooski, VT, USA) at 530/590 nm. Fluorescence values of each triplicate were averaged and fitted to the standard curve to calculate free fatty acid concentrations. Significant differences (*p* < 0.05) between treatment groups were determined using analysis of variance and treatment by age effects were determined using two-way ANOVA (SAS, Cary, NC, USA).

### 2.4. β-Hydroxybutyrate (Ketone Body) Assay

Plasma β-Hydroxybutyrate levels were measured using a β-Hydroxybutyrate (Ketone Body) Colorimetric assay kit (Cayman Chemical), following the manufacturer’s instructions. Briefly, plasma from six males in each group was assayed in triplicate. Plasma was diluted 1:10 in assay buffer. To 50 µL of diluted plasma, 50 µL of developer solution was added and samples were incubated at 25 °C for 30 min. Absorbance was measured at 450 nm on a BioTek Synergy HT plate reader (BioTek). Absorbance values of each triplicate were averaged and fitted to the standard curve to calculate β-Hydroxybutyrate concentrations. Significant differences (*p* < 0.05) between treatment groups were determined using analysis of variance and treatment by age effects were determined using two-way ANOVA (SAS).

### 2.5. Triglyceride Assay

Plasma triglyceride levels were measured using a Triglyceride Colorimetric assay kit (Cayman Chemical), following the manufacturer’s instructions. Briefly, plasma from six males in each group was assayed in duplicate. To 10 µL of plasma, 150 µL of enzyme mixture was added and samples were incubated room temperature for 15 min. Absorbance was measured at 540 nm on a BioTek Synergy HT plate reader (BioTek). Absorbance values of each duplicate were averaged and fitted to the standard curve to calculate triglyceride concentrations. Significant differences (*p* < 0.05) between treatment groups were determined using analysis of variance and treatment by age effects were determined using two-way ANOVA (SAS).

### 2.6. Glucose Assay

Plasma glucose levels were measured using a Glucose Colorimetric assay kit (Cayman Chemical), following the manufacturer’s instructions. Briefly, plasma from six males in each group was assayed in duplicate. Plasma was diluted 1:10 in assay buffer. To 15 µL of diluted plasma 85 µL of diluted assay buffer was added, followed by 100 µL of enzyme mixture. Samples were incubated for 10 min at 37 °C. Absorbance was measured at 515 nm on a BioTek Synergy HT plate reader (BioTek). Absorbance values of each duplicate were averaged and fitted to the standard curve to calculate glucose concentrations. Significant differences (*p* < 0.05) between treatment groups were determined using analysis of variance and treatment by age effects were determined using two-way ANOVA (SAS).

### 2.7. RNA Isolation and cDNA Synthesis

Total RNA was isolated from 50 mg of liver tissue using Tri-Reagent (Sigma-Aldrich St. Louis, MO, USA) following the manufacturer’s instructions with the exception that the RNA was precipitated overnight at −20 °C. Total RNA was DNase-treated using a TURBO-DNA free kit (Thermo Fisher Scientific, Waltham, MA, USA) following the manufacturer’s instructions. RNA quality was assessed using agarose electrophoresis. One microgram of DNase-treated total RNA per sample was reverse transcribed using a miScript II RT kit (Qiagen, Germantown, MD, USA) following the manufacturer’s instructions.

### 2.8. Real-Time Quantitative PCR

For mRNAs, forward and reverse primers were designed using primer-BLAST (https://www.ncbi.nlm.nih.gov/tools/primer-blast/) and, for miRNAs, the forward primer consisted of the mature miRNA sequence and the reverse primer used was the miScript universal primer (Qiagen). Ribosomal protein L4 (*RPL4*) and the small nucleolar RNA, *snoU83B*, were used as housekeeping genes for mRNA and miRNA normalization, respectively. All primer sequences are provided in Table S2. For small RNA expression analysis, each reaction contained 10 ng of cDNA, 500 nmol of gene-specific forward primer, 1× Universal miScript primer (Qiagen) and 1× iQ SYBR Green Supermix (Bio-Rad, Hercules, CA, USA). The following PCR conditions were used: 95 °C for 5 min, followed by 40 cycles of 95 °C for 10 s, then 58 °C for 20 s. All reactions were performed in duplicate. Gene specific amplification was confirmed using melting curve analysis. The same conditions were also used for gene expression (mRNA) analysis with the exception that 500 nmol of both a gene-specific forward primer and a gene-specific reverse primer was used. Threshold cycle (Ct) values normalized to the expression levels of *snoU83B* (for miRNAs) or *RPL4* (mRNAs). Significant (*p* < 0.05) differences in expression were determined using analysis of variance.

### 2.9. Yeast One-Hybrid Screening of the FASN Promoter

Yeast one-hybrid analyses were carried out using the Matchmaker Gold yeast one-hybrid system (Clontech, Mountain View, CA, USA) as directed by the manufacturer. The upstream region (~4 kb) of chicken *FASN* was obtained from Ensembl (http://useast.ensembl.org/index.html), and promoter-like elements upstream of *FASN* were predicted using PROSCAN (https://www-bimas.cit.nih.gov/molbio/proscan/). The region containing all potential regulatory elements (~350 bp) was cloned from Ross 708 genomic DNA into the pAbAi vector using KpnI and XhoI and sequenced. A bait strain containing the *FASN* promoter cassette was generated following the manufacturer’s instructions. Briefly, Y1H Gold yeast were transformed with 1 µg of linearized (using BstBI) pAbAi-gga-FASN-pro vector and yeast with positive cassette integration were selected using SD-Ura media. Y1H Gold-pAbAi-FASN-pro yeast were then tested on SD-Ura containing a range of Aureobasidin A (Aba) concentrations to determine the optimal Aba concentration for library screening (350 ng/mL). The cDNA library was produced from liver tissues. For library construction, total RNA was purified from liver tissue of Ross 708 males (n = 6) and pooled (8 µg each) from E18, E20, D0, D1 and D3. For cDNA production, mRNA was purified using a NucleoTrap mRNA kit (Clontech). One microgram of mRNA from each sample was pooled, and one microgram of pooled mRNA was used for reverse-transcription using SMART RT (Clontech). SMART cDNA was then used in long-distance PCR to produce a double-stranded cDNA library following the manufacturer’s instructions. Library quality was assessed using gel electrophoresis. The library was purified using a CHROMA SPIN + TE-400 column (Clontech) and concentrated (ethanol/sodium acetate precipitation) as directed by the manufacturer. The cDNA library (5.2 µg) was transformed into the Y1H Gold-pAbAi-FASN-pro yeast following the manufacturer’s instructions and screened on SD-Leu media containing 350 ng/mL Aba. Positive colonies were further selected by re-plating (3×) on SD-Leu-350 ng/mL Aba. Approximately 2.5 million yeast colonies were screened.

## 3. Results

### 3.1. Impact of Delayed Feeding on Chick Weights and Plasma Metabolic Status Indicators

**Figure 1:**
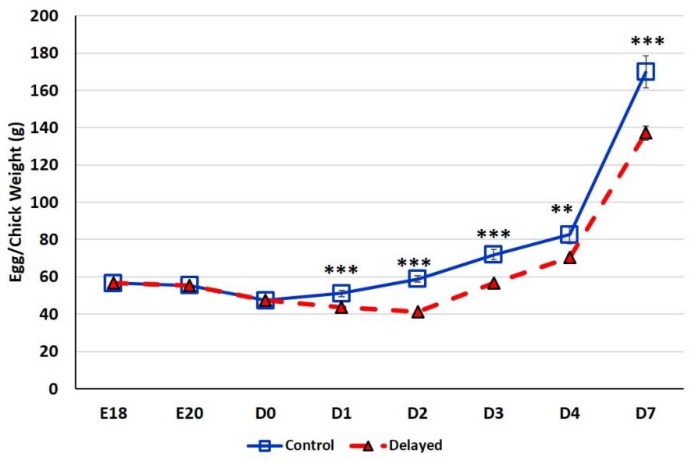
Body weight gain (g) of fed from hatch and delayed fed broiler chicks. For uniformity, eggs were weighed prior to blood collection therefore at embryonic days (E) E18 and E20 mean egg weights are provided (n = 6 males). At day hatch (D) D0–D7, chick mean weights are provided (n = 6 males/treatment). Error bars denote standard deviations. Significant (*p* < 0.05) differences between treatment groups were determined using analysis of variance. ** *p* < 0.001, *** *p* < 0.0001 denote significant differences between fed at hatch chicks and chicks with delayed access to feed for 48 h.

At E18, the average egg weight of the six male embryos selected for analysis was 56.64 ± 0.27 g (Figure 1). At E20, the average egg weight dropped slightly to 55.44 ± 0.20 g. At D0 the average chick weight was 47.36 ± 0.37 g. At D1, fed chicks weighed significantly more (*p* < 0.0001) with an average weight of 51.12 ± 1.75 g than delayed fed chicks which weighed an average of 43.65 ± 0.56 g. By D2 fed chicks weighed ~30% more (*p* < 0.0001) at 58.92 ± 1.58 g than delayed fed chicks, the average weight of this group dropped to 41.27 ± 0.48 g. At D3, chick fed since hatch weighed an average of 71.93 ± 2.67 g, while chicks delayed access to feed for 48 h weighed significantly less (*p* < 0.0001) at 56.71 ± 0.91 g. By day 4 post-hatch, chicks receiving feed since hatch weighed an average of 82.60 ± 4.86 g, while the delayed chicks weighed 70.44 ± 1.50 g (*p* < 0.001). On D7, chicks delayed access to feed for 48 h still weighed significantly less (*p* < 0.0001) at 137.05 ± 3.73 g than chicks receiving feed since hatch which weighed 169.90 ± 8.57 g. Chicks delayed access to feed for 48 h gained 27% less weight than their normal fed counterparts during the first week post-hatch. Comparable weights (*p* > 0.05) between control birds and delayed fed birds was not reached until D16.

**Figure 2:**
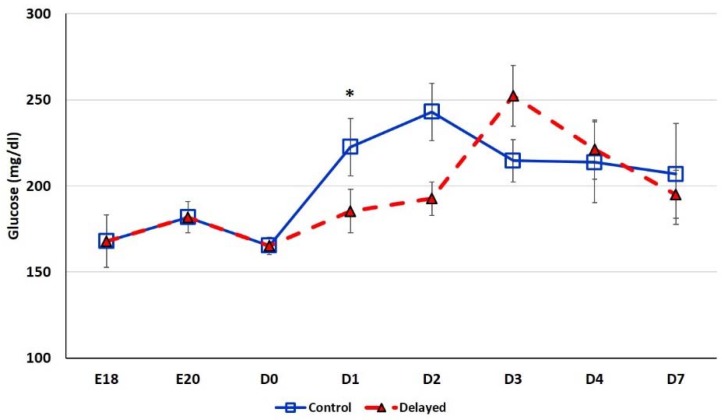
Plasma glucose levels (mg/dL) of fed from hatch and delayed fed broiler chicks. Mean (n = 6 males/treatment) plasma glucose levels. Glucose was measured using a glucose colorimetric assay kit (Cayman Chemical). Error bars denote standard deviations. Significant (*p* < 0.05) differences between treatment groups were determined using analysis of variance. * *p* < 0.05.

**Figure 3:**
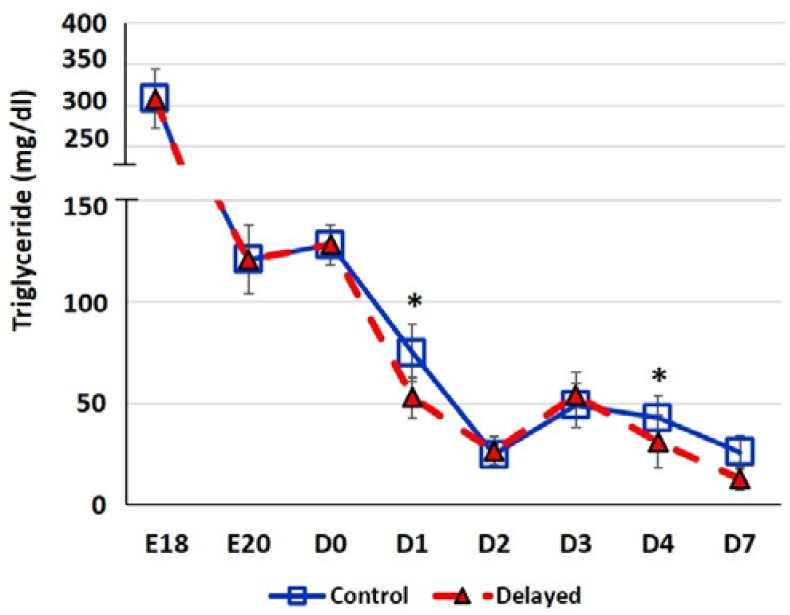
Plasma triglyceride levels (mg/dl) of fed from hatch and delayed fed broiler chicks. Mean (n = 6 males/treatment) plasma triglyceride levels. Triglyceride was measured using a triglyceride colorimetric assay kit (Cayman Chemical). Error bars denote standard deviations. Significant (*p* < 0.05) differences between treatment groups were determined using analysis of variance. * *p* < 0.05.

**Figure 4:**
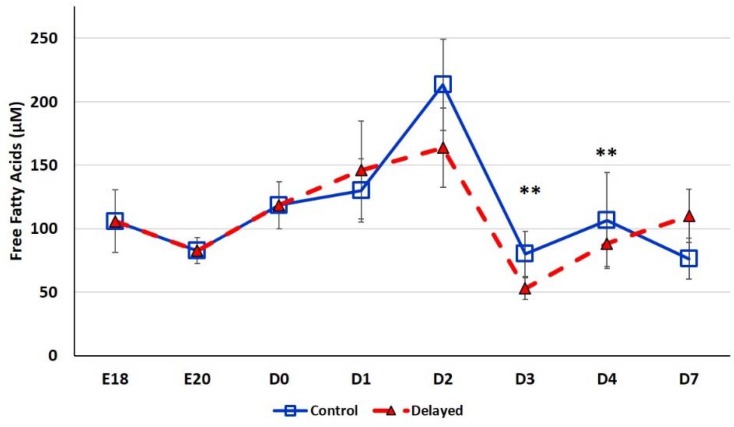
Plasma free fatty acid levels (µM) of fed from hatch and delayed fed broiler chicks. Mean (n = 6 males/treatment) plasma free fatty acid levels. Free fatty acids were measured using a free fatty acid fluorometric assay kit (Cayman Chemical). Error bars denote standard deviations. Significant (*p* < 0.05) differences between treatment groups were determined using analysis of variance. ** *p* < 0.001 denotes a significant difference between fed at hatch chicks and chicks delayed access to feed for 48 h.

**Figure 5:**
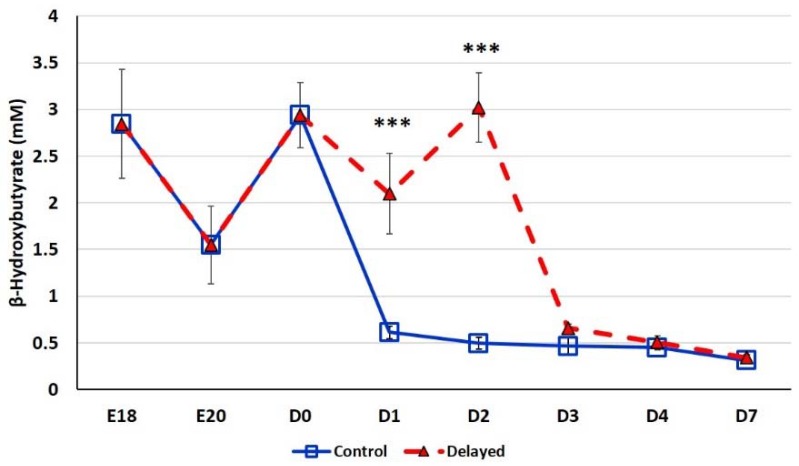
Plasma β-hydroxybutyrate levels (mM) of fed from hatch and delayed fed broiler chicks. Mean (n = 6 males/treatment) plasma β-hydroxybutyrate levels. β-hydroxybutyrate was measured using a β-hydroxybutyrate (ketone body) colorimetric assay kit (Cayman Chemical). Error bars denote standard deviations. Significant (*p* < 0.05) differences between treatment groups were determined using analysis of variance. *** *p* < 0.0001 denotes a significant difference between fed at hatch chicks and chicks delayed access to feed for 48 h.

Chicks delayed access to feed for the first 48 h post-hatch had lower plasma glucose levels than chicks receiving feed at hatch (Figure 2). Within 24 h of feeding, delayed feed chicks had slightly higher plasma glucose levels, but quickly equalized to fed-from-hatch birds. However, no age or treatment affects were detected (*p* > 0.05). Plasma triglyceride levels tended to be higher in control birds and was significantly impacted (*p* < 0.01) by both age and delayed feeding (Figure 3). Free fatty acids were slightly higher in control birds, particularly at D3 and D4 (Figure 4), however two-way ANOVA analysis only found a significant impact by age (*p* < 0.0001), but no delayed feeding effect (*p* > 0.05). Delayed feeding markedly impacted plasma β-hydroxybutyrate (ketone bodies) levels (Figure 5). Two-way ANOVA indicated that both age and delayed feeding significantly (*p* < 0.0001) impacted plasma β-hydroxybutyrate levels. At D1, delayed fed chicks had three-fold higher β-hydroxybutyrate levels and at D2 had six-fold higher levels. Within 24 h of feed consumption, β-hydroxybutyrate levels of delayed fed chicks reached comparable levels to chicks fed since hatch. Until feeding, the β-hydroxybutyrate levels of delayed chicks were more similar to E18 embryos than their age-matched normal fed counterparts.

### 3.2. Impact of Delayed Feeding on Hepatic miRNA Expression

**Figure 6:**
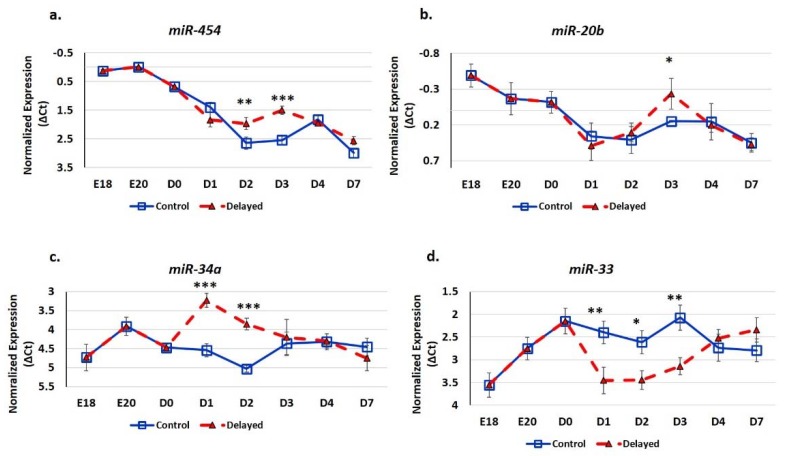
Delayed feeding of peri-hatch broiler chicks alters hepatic miRNA expression. (**a**–**d**) Real-time quantitative PCR (RT-qPCR) was utilized to measure the expression of hepatic miRNA expression. Values are shown as the mean ΔCt (n = 6 males) for each treatment group (fed at hatch or delayed) at each time point. MiRNA expression levels were normalized to *snoU83B* levels. Error bars denote standard deviations. Significant (*p* < 0.05) differences in expression were determined using analysis of variance. * *p* < 0.05, ** *p* < 0.01, *** *p* < 0.001 denote significant differences between fed at hatch chicks and chicks with delayed access to feed for 48 h.

We utilized real-time quantitative PCR (RT-qPCR) analysis to determine the impact of delayed feeding on the expression of hepatic miRNA (Figure 6). We found that delaying access to feed for 48 h post-hatching significantly (*p* < 0.05) increased the hepatic expression of *miR-454*, *miR-20b*, and *miR-34a* and decreased the expression of *miR-33*. The miRNA *miR-454* was most highly expressed in the embryonic liver (Figure 6a). Chicks with delayed access to feed for 48 h had 1.6-fold higher hepatic *miR-454* expression at D2 and 2.1-fold higher expression at D3 (Figure 6a). By D4, hepatic levels of *miR-454* were comparable between delayed fed chicks and chicks fed at hatch. Similar to *miR-454*, *miR-20b* had higher expression in the embryonic liver (Figure 6b). Delayed fed chicks had higherhepatic expression of *miR-20b* and, at D3, in delayed fed chicks, hepatic levels of *miR-20b* were more similar to embryonic levels than to their age-matched normal fed counterparts (Figure 6b). The hepatic expression of *miR-34a* was over two-fold higher in delayed fed chicks at D1 and D2, but reached similar levels to fed from hatch chicks within 24 h of feed consumption (Figure 6c). *miR-33* level was approximately two-fold lower in the livers of delayed fed chicks at D1, D2, and D3, but had similar levels to fed from hatch chicks at D4 and D7 (Figure 6d).

### 3.3. Impact of Delayed Feeding on Hepatic mRNA Expression

**Figure 7:**
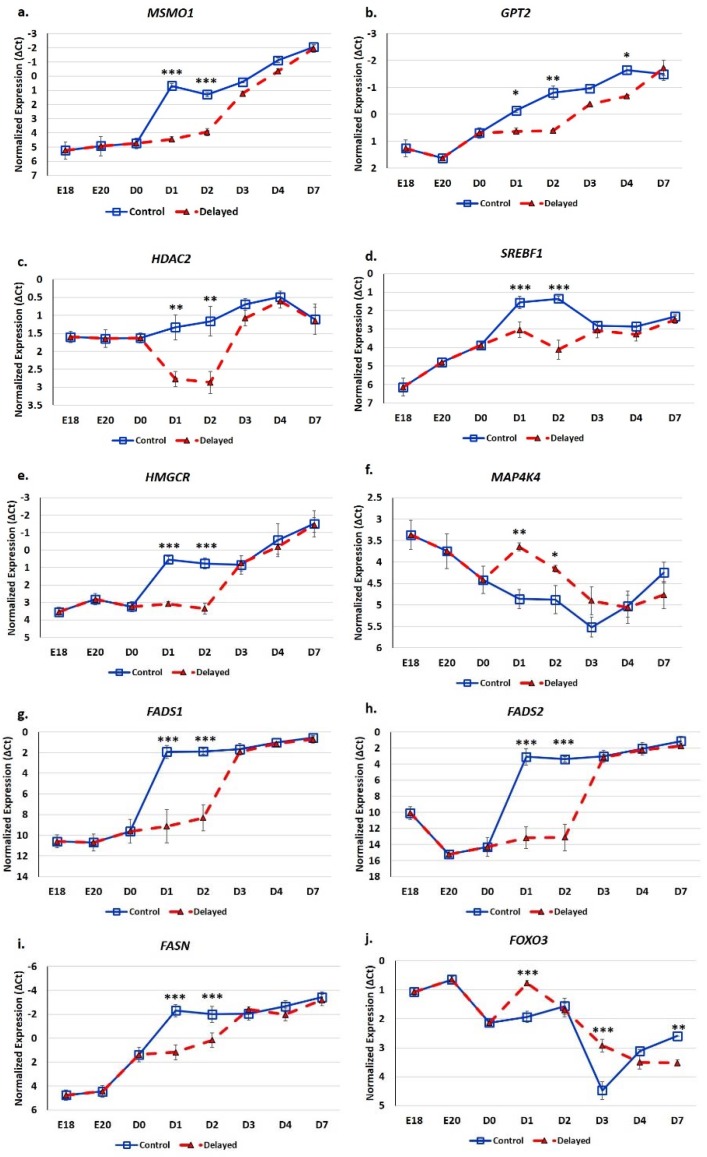
Delayed feeding of peri-hatch broiler chicks alters hepatic expression of glucose metabolic and lipid metabolic genes. (**a**–**j**) RT-qPCR was utilized to measure the expression of hepatic mRNA expression. Values are shown as the mean ΔCt (n = 6 males) for each treatment group (fed at hatch or delayed) at each time point. mRNA expression levels were normalized to RPL4 levels. Error bars denote standard deviations. Significant (*p* < 0.05) differences in expression were determined using analysis of variance. * *p* < 0.05, ** *p* < 0.01, ***p < 0.001 denote significant differences between fed at hatch chicks and chicks with delayed access to feed for 48 h.

RT-qPCR analysis of glucose and lipid metabolic genes indicates these processes are significantly impacted by delaying feeding for 48 h post-hatching (Figure 7). Hepatic expression of *MSMO1* was 13.8-fold lower at D1 (*p* < 0.001) and 6.2-fold lower at D2 (*p* < 0.001) in delayed fed chicks compared to fed from hatch chicks (Figure 7a). *GPT2* had 2–3-fold lower hepatic expression in delayed fed chicks at D1 (*p* < 0.05), D2 (*p* < 0.01) and D4 (*p* < 0.05) (Figure 7b). *HDAC2* levels were approximately three-fold lower in the livers of delayed fed chicks at D1 (*p* < 0.01) and D2 (*p* < 0.01), but quickly matched levels from fed from hatch chicks, upon feed consumption (Figure 7c). At D1, *SREBF1* was expressed 2.8-fold less (*p* < 0.001) in the livers of delayed fed chicks and was further repressed at D2, at 6.7-fold lower (*p* < 0.001) (Figure 7d). *HMGCR* hepatic expression was about six-fold lower (*p* < 0.001) in delayed fed birds until the initiation of feed consumption (Figure 7e). *MAP4K4* was significantly higher expressed in the livers of delayed fed chicks at D1 (*p* < 0.01) and D2 (*p* < 0.05) and still slightly higher at D3 (Figure 7f). Delayed fed chicks had very low hepatic levels of *FADS1* and *FADS2*, similar to embryonic levels, until feed consumption, when both quickly reached the levels of fed from hatch chicks (Figure 7g,h). On both D1 and D2, *FADS1* and *FADS2* hepatic levels were several hundred-fold lower in delayed fed chicks. *FASN* had significantly lower levels in the livers of delayed fed chicks on D1 and D2 (Figure 7i), but, similar to *FADS1* and *FADS2*, its levels matched fed from hatch chicks within 24 h of feed consumption. *FOXO3* hepatic expression was higher in delayed fed chicks on D1 (*p* < 0.001) and D3 (*p* < 0.001) (Figure 7j). On D1, *FOXO3* levels were more similar between embryos and delayed fed chicks than fed from hatch chicks (Figure 7j).

### 3.4. Yeast One-Hybrid Identification of Regulators of Hepatic FASN Expression

To identify potential hepatically-expressed transcriptional regulators affected by delayed feeding, we utilized a yeast one-hybrid screen to identify factors that can recognize the *FASN* promoter. *FASN* was chosen as it is a major member of lipogenic metabolic processes by catalyzing the formation of long chain fatty acids and its hepatic expression was found to be highly sensitive to feed consumption (Figure 7i). Potential regulatory proteins, with multiple hits in the hepatic prey library, which likely regulate the hepatic expression of *FASN* in chickens, include *SRF* (8 clones), *PKNOX1* (10 clones) and *HDAC2* (16 clones).

## 4. Discussion

Although the physiological processes involved in the metabolic switch in peri-hatch chicks have been somewhat studied, the underlying molecular mechanisms governing them is unknown. We recently have shown that miRNAs are dynamically expressed during the peri-hatching period and that these miRNAs target a number of metabolically important genes and pathways [14]. These targeted pathways include those associated with lipolysis, lipid oxidation, lipogenesis, and glucose metabolism. To further elucidate the roles of miRNAs and other molecular regulatory systems in the metabolic switch, we utilized a delayed feeding regime to retard the metabolic shift in broilers. Birds in the delayed fed group gained less weight, had lower plasma glucose levels and had much higher β-hydroxybutyrate levels then chicks fed at hatch, with more comparable levels to embryonic chicks (Figure 1, Figure 2 and Figure 4), indicating that their metabolic transition was delayed by delayed feeding.

It has long been established that lack of intake of a carbohydrate-based food source by peri-hatch chicks increases the breakdown of yolk fats for energy production and often results in ketosis [15]. Ketone bodies are generated from lipid oxidation as a result of using stored fats for energy production rather than glucose [4]. The high production of ketone bodies in late stage embryonic chicks is thought to provide energy to the developing tissues, particularly muscle. This allows the developing embryo to preserve the small amounts of carbohydrates for biosynthetic processes rather than use it as an energy source [4]. In the present study, we also observed this phenomenon; here delayed fed chicks had much higher plasma ketones than their normal fed counterparts (Figure 5). However, within 24 h of feeding, delayed fed chicks had a significant decline in plasma ketone bodies and matched levels of normal fed chicks (Figure 5). At D1, delayed fed chicks had lower plasma triglycerides (Figure 3) than fed chicks, further suggesting that fasted chicks must heavily rely on yolk triglyceride stores. It has been shown that high levels of ketone bodies reduce HMG-CoA reductase (HMGCR) activity [16]. We found that hepatic HMG-CoA reductase mRNA levels were significantly lower in delayed fed chicks until the initiation of feed consumption (Figure 7). This indicates that the delayed fed chicks were forced to utilize oxidation of residual yolk lipids as their main energy source, however, were able to quickly begin to utilize feed-based carbohydrate energy sources upon the initiation of feeding.

The hepatic expression of *miR-454* was higher in delayed fed chicks (Figure 6), while two of its targets *MSMO1* and *GPT2* displayed lower hepatic expression in these chicks (Figure 7). MSMO1 is a sterol-C4-methyl oxidase involved in cholesterol biosynthesis. We previously showed that *MSMO1* is also regulated by *miR-20b*, which similar to *miR-454* is higher expressed in the livers of delayed fed chicks. As discussed above, delayed fed chicks also had significantly lower hepatic levels of *HMGCR* (Figure 7). HMGCR is also involved in cholesterol biosynthesis, as statins, drugs that inhibit HMGCR function, reduce cholesterol synthesis (reviewed by [17]). These results suggest that cholesterol synthesis is not fully functional until the initiation of feed consumption. Furthermore, it is likely that miRNAs are important regulators of cholesterol biosynthetic pathways in chickens. GPT2 is involved in gluconeogenesis. In humans, GPT2 can serve as a biomarker of insulin resistance and is elevated in diabetic patients [18]. GPT2 levels are also positively correlated with hepatic glucose output [18]. In the present study, delayed fed chicks had significantly lower plasma glucose levels until the initiation of feeding, after which there was a sharp spike in plasma glucose levels (Figure 2). These results suggest that miRNA-mediated regulation may serve to limit production of gluconeogenesis related enzymes in newly hatched chicks until a dietary source for glucose production is available.

We previously found that hepatic *miR-20b* expression can be regulated by *FOXO3* [14]. Here, we found that *FOXO3* expression is 2.9-fold higher expressed in the livers of delayed fed chicks at D3 post-hatch compared to chicks fed on D0, and *miR-20b* hepatic expression in delayed fed chicks is also significantly higher (Figure 6b and Figure 7j). This suggests that the increased expression of *miR-20b* in the livers in delayed fed chicks is due to, at least in part, increased FOXO3 expression. Increased FOXO3 expression helps protect cells from oxidative stress (reviewed by [19]). Oxidation of lipids produces ketone bodies as a byproduct. Therefore, high plasma levels of ketone bodies are an indicator that fats rather than glucose are being utilized for energy. The much higher levels of ketones in delayed fed chicks indicates that as expected the oxidation of yolk lipid stores are their main source for energy production. Thus, it is likely that they may be more susceptible to hepatic oxidative stress. In mammals consuming a high fat diet and/or experiencing non-alcoholic fatty liver disease, hepatic oxidative stress also occurs [20,21]. Therefore, the higher levels of *FOXO3* expression in delayed fed chicks may be induced to protect against oxidative stress due to excess lipid oxidation. In fact, in mammals, in addition to serving as alternative energy source, β-hydroxybutyrate (ketone bodies) can also serve as a signaling molecule of oxidative pressure [22]. It was shown in a failing heart rodent model that β-hydroxybutyrate is elevated and this elevation leads to increased FOXO3 expression [22]. It was further shown that FOXO3 repressed the expression of class I histone deacetylases, including HDAC2, and this repression contributed the protective mechanism from oxidative stress [22]. FOXO3 is transcriptional regulator which can function as both an activator and repressor of transcription [23]. We previously found that hepatic *FOXO3* hepatic expression is higher in the late stage embryo than in newly hatched birds [14]. As mentioned above, here we found that hepatic *FOXO3* expression was higher in delayed fed birds [14]. Conversely, *miR-20b*, which we previously found to be regulated by FOXO3 [14], was also upregulated in the livers of delayed fed birds (Figure 6). As discussed above, the *miR-20b* target *MSMO1* was lower in the livers of delayed fed birds (Figure 7). MSMO1 is one of the main enzymes responsible for cholesterol biosynthesis [24]. As discussed above, FOXO3 has been shown to negatively regulate the expression of HDAC2 in response to elevated β-hydroxybutyrate, as a protective mechanism from oxidative stress [22]. It has also been demonstrated that HDAC2 expression can be post-transcriptionally regulated by *miR-34a* [25]. In mammals, *miR-34a* is considered an important mediator of hepatic lipid homeostasis [26]. In the present study, hepatic expression of *miR-34a* was higher in delayed fed birds (Figure 6). Interestingly, FOXO3 also been shown to function as an activator of *miR-34a* expression [27]. Taken together, our results suggest a complementary transcriptional and post-transcriptional system that works in concert to balance the energy needs of the developing chick and at the same time protects hepatocytes from the oxidative stresses associated with the oxidation of yolk lipids. Physiological data in conjunction with the gene expression data implicate a regulatory system in which accumulation of ketone bodies (β-hydroxybutyrate) from oxidation of yolk-derived lipids induces the expression the transcriptional regulator FOXO3. FOXO3 in turn either induces or represses expression of genes and miRNAs to maintain a balance of sufficient energy production and minimization of damage from its associated oxidative stresses.

SREBF1 is induced by high fat diets in rodents (reviewed by [28]). SREBF1 then, in turn, induces the expression of enzymes involved in de novo lipogenesis, monounsaturated fatty acid synthesis (MUFA), and triglyceride synthesis and storage. Thus, SREBF1 is thought to be a master regulator of fatty acid synthesis pathways. In the present study, we found that hepatic *SREBF1* expression was significantly lower in delayed fed chicks until the initiation of feed consumption (Figure 7). At D2, delayed fed chicks had significantly lower plasma free fatty acids levels. This suggests that delayed fed chicks quickly utilized their fat stores for energy production and had minimal de novo fatty acid synthesis, likely due to reduced expression of SREBF1 and its downstream pathways. A member of the mitogen-activated protein kinase family, MAP4K4, has been shown to be an inhibitor of lipogenesis, in part, by repressing SREBF1 expression [29]. We previously reported that hepatic *MAPK4K4* expression was higher in embryos than in post-hatch chicks [14]. Here, we found that hepatic *MAPK4K4* expression was higher in delayed fed chicks, which likely contributes to the lower expression of SREBF1 observed in the present study (Figure 7). *miR-33* is a well-known regulator of lipid metabolism in vertebrates and was recently shown to inhibit hepatic fatty acid oxidation in chickens [30] and is predicted to regulate the expression of chicken *MAPK4K4* (www.targetscan.org). Hepatic expression of *miR-33* was significantly lower in delayed fed birds (Figure 6), further supporting a role for *miR-33* in regulating hepatic lipid metabolism and suggesting it is an important post-transcriptional mediator of the metabolic switch in chickens.

FASN is a SREBF1-regulated enzyme that serves to convert acetyl-CoA into long chain fatty acids. We previously showed that *FASN* mRNA levels in the embryonic and day of hatch chick are quite low then quickly increase upon feeding. Here, we found that hepatic *FASN* levels of delayed fed chicks were much lower than normal fed birds (Figure 7). However, upon feed consumption hepatic *FASN* levels of delayed feed chicks quickly (within 24 h) reached levels similar to their normal fed counterparts (Figure 7). To further elucidate the regulation of *FASN* expression during the metabolic switch, we utilized a yeast one-hybrid system to screen a broiler hepatic cDNA library for potential transcriptional regulators. Identified transcriptional regulators included *HDAC2*, *PKNOX1*, and *SRF*. SRF has been shown to mediate the response to glucose by regulating a number of glucose related enzymes such as LXRB [31]. Small interfering RNA-mediated reduction of SRF in a rat insulinoma cell line partially inhibited the ability of these cells to respond to glucose [31]. The transcriptional regulator PKNOX1 is higher in the livers of diabetic and non-alcoholic fatty liver disease patients [32]. It was found that PKNOX1 could be regulated by *miR-17* family members, including *miR-17* and *miR-20a* [32]. Overexpression of *miR-17* and *miR-20a* in human hepatocytes increased the sensitivity of these cells to insulin and also decreased triglyceride and lipid accumulation [32]. As discussed above, HDAC2 is a histone deacetylase that regulates many different biological processes, including anti-oxidative mechanisms and gluconeogenesis [33]. Treatment of the human hepatoma cell line HepG2 with an HDAC2-specific siRNA reduced the glucose metabolic potential of these cells [33]. It is reported that, upon feeding of fasted mice, HDAC2 binds to the promoter of CYP7A1 (an enzyme that converts cholesterol to bile acids) to increase cholesterol biosynthesis [34]. Interestingly, as discussed above, HDAC2 expression can be repressed by FOXO3 [22] and can also be regulated by *miR-34a* [27]. It has also been shown that elevated β-hydroxybutyrate can induce FOXO3, as part of the anti-oxidant protective mechanism [22]. We found that β-hydroxybutyrate, *FOXO3*, and *miR-34a* levels were higher in delayed fed birds (Figure 5, Figure 6 and Figure 7), while *HDAC2* and *FASN* levels were lower (Figure 7). Overall, the potential for FASN expression to be regulated by HDAC2, SRF, and PKNOX1 suggests that a complex interconnected regulatory network is involved in controlling lipid and glucose molecular pathways during the metabolic transition in peri-hatch chicks.

**Figure 8:**
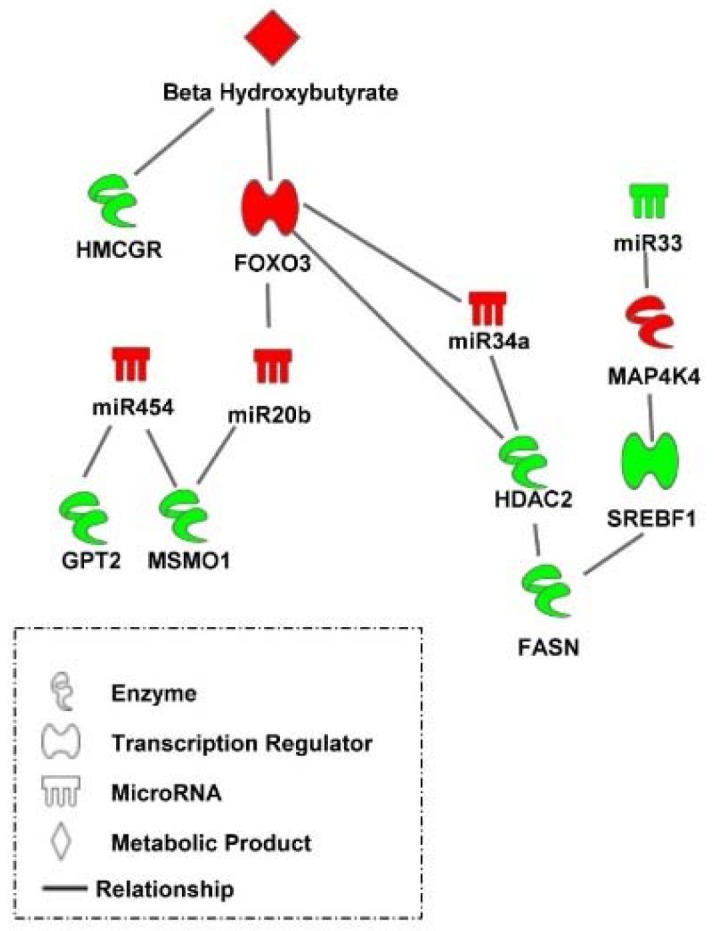
A transcriptional and post-transcriptional regulatory system mediating the hepatic metabolic switch in chickens. A delayed feeding regime to retard the metabolic switch in broilers uncovered a complex transcriptional and post-transcriptional hepatic regulatory system which is triggered by nutritional/physiological cues to facilitate the metabolic transition in peri-hatch chickens. Red molecules have higher levels in broiler chicks with delayed access to feed for 48 h post-hatching compared to their fed from hatch counterparts and green molecules had lower levels in delayed fed chicks. Pathway was created using Ingenuity Pathway Analysis.

Taken together, the results of the present study indicate that a complex transcriptional and post-transcriptional hepatic regulatory system is triggered by nutritional/physiological cues to facilitate the metabolic transition in peri-hatch chickens (Figure 8). For example, high levels of β-hydroxybutyrate resulting from oxidation of yolk fatty acids in embryonic chicks and newly hatch chicks prior to feeding, maintains lower levels of lipogenic factors (e.g., HMGCR) and elevated levels of transcriptional regulators (e.g., FOXO3) and post transcriptional regulators (e.g., *miR-454*, *miR-20b*, and *miR-34a*) which mute lipogenic processes (e.g., FASN, MSMO1, and SREBF1) and concurrently protect hepatocytes from the stresses of lipid oxidation (e.g., HDAC2).

## 5. Conclusions

A complex molecular regulatory system underlies the hepatic metabolic program switching that occurs in young chicks transitioning from embryonic to post-hatch life. The present study demonstrated that many transcriptional and post-transcriptional, including miRNA and regulatory factors, mediate this switch. Furthermore, this study suggests the molecular program of delayed fed chicks catches up quickly upon feeding. However, the physiological translation of this is more gradual, indicating long term consequences on growth and development in delayed fed chicks. Here, we identified some of the central molecular mechanisms of the metabolic switch in chickens (Figure 8). This information will likely be beneficial in developing more targeted strategies to improve the metabolic transition and thus growth and development of poultry. For example, a rapidly growing field of interest in the poultry industry is the enhancement of neonatal chick development by administering nutrients in ovo [35,36,37,38]. It has been established that newly hatched chicks have an under-developed digestive system and cannot process feed as efficiently as older birds [35]. Studies have shown that admiration of nutrients such as vitamins, minerals, and carbohydrates to late stage embryos (i.e., in ovo feeding) can improve digestive system maturation and feed efficiencies upon hatching and have long-term benefits on metabolic health and overall growth [35,36,37,38]. The results of the present study could be useful in the development of better focused in ovo feeding strategies by providing insight into the most critical metabolic pathways to target for enhancement of early life metabolic programming.

## Supplementary Materials

The following are available online at https://www.mdpi.com/2073-4425/10/4/272/s1, Table S1: Starter Feed Composition; Table S2: Primers used in this study.
