# Supplementary material for: Delayed Feeding Alters Transcriptional and Post-Transcriptional Regulation of Hepatic Metabolic Pathways in Peri-Hatch Broiler Chicks
Source: Genes (Basel). 2019 Apr 3;10(4):272. doi: 10.3390/genes10040272 (PMC6523616; doi:10.3390/genes10040272)
Supplement: Supplementary file 1 [file genes-10-00272-s001.zip › genes-459370 supplementary.pdf]

Supplemental Table S1. Starter feed composition (Southern States)

Active Drug Ingredient	
Lasalocid	113.00 g/ton
Guaranteed Analysis	
Crude Protein (min)	28.00%
Lysine (min)	1.40%
Methionine (min)	0.75%
Crude Fat (min)	4.00%
Crude Fiber (max)	4.00%
Calcium (min)	1.00%
Calcium (max)	1.50%
Phosphorus (min)	0.75%
Salt (min)	0.20%
Salt (max)	0.70%
Sodium (min)	0.15%
Sodium (max)	0.65%
