# Supplementary material for: Delayed Feeding Alters Transcriptional and Post-Transcriptional Regulation of Hepatic Metabolic Pathways in Peri-Hatch Broiler Chicks
Source: Genes (Basel). 2019 Apr 3;10(4):272. doi: 10.3390/genes10040272 (PMC6523616; doi:10.3390/genes10040272)
Supplement: Supplementary file 1 [file genes-10-00272-s001.zip › genes-459370 supplementary2.pdf]

Supplemental Table S2. Primers used in this study

miR-454	TAGTGCAATATTGCTTATAGGGT
miR-20b	CAAAGTGCTCATAGTGCAGGTAG
miR-33	GTGCATTGTAGTTGCATTGC
miR-34a	TGGCAGTGTCTTAGCTGGTTGTT
snoU83B	ACCATGGAATAAGCGCTGGGCA

FADS1-F	ACGCCACGGATCCTTTCATAG
FADS1-R	AAGCTGGGTTGATCCGGTG
FADS2-F	TCTTAATGGGGAGGGAACAGGT
FADS2-R	ACCAGCTTCTTGCATTTTCACA
FASN-F	GGCTACACACTAGTTGGCACT
FASN-R	CACTGTGTTCCCATGCCTGA
FOXO3-F	TGAATGTGGGGAACTTCACTGG
FOXO3-R	TGAGGGCCTGTTTGCCCATT
GPT2-F	TCCTTCGTCAGGTAGTGGCA
GPT2-R	CAACCCTGCAAGATCCGTCT
HADC2-F	CAGATGCAGGCCATTCCTGA
HADC2-R	CAGATGCTCGAATAGAAATGCGT
HMGCR-F	GGAGTGCGACAACT
HMGCR-R	ACACACAAGCTGGA
MAP4K4-F	GCAGGCCATGTTACTGGAATAC
MAP4K4-R	TGACAGGAGGTAGGCTTGCT
MSMO1-F	CATCCTTGGAGCTGGCTTCTT
MSMO1-R	TCATAGCCACTGTGCACATCAA
RPL4-F	GCACCGCAGAGTGAACGTAA
RPL4-R	CTCCTCAATGCGGTGACCTTT
SREBF1-F	CTCAGCGACATCGACGACAT
SREBF1-R	GACACGGTGCTGTCAGGG
